# Induced RNA Interference Can Suppress Persistent *Drosophila* A Virus (DAV) Infection in Cultured Drosophila Melanogaster S2 Cells

**DOI:** 10.3390/ncrna12040028

**Published:** 2026-07-30

**Authors:** Wiebke Kochendörfer, Klaus Förstemann

**Affiliations:** Gene Center and Department of Biochemistry, Ludwig-Maximilians-Universität München, Feodor-Lynen-Strasse 25, 81377 München, Germany; w.kochendoerfer@lmu.de

**Keywords:** *Drosophila* A virus, persistent infection, RNA interference (RNAi), S2 cell lines, double-stranded RNA (dsRNA), viral suppression

## Abstract

**Background**: The RNA interference (RNAi) pathway is a highly conserved antiviral mechanism in eukaryotes, including insects such as fruit flies. Many viruses have therefore evolved mechanisms that protect their transcripts and/or genomes; how effectively RNAi can act is thus a question that must be answered case-by-case. **Methods**: We demonstrate that the RNAi machinery can successfully combat *Drosophila* A virus (DAV) in persistently infected *Drosophila melanogaster* S2 cell lines, but only when supported with exogenous triggers. Since such an infection poses a challenge to the fitness of cells and the reproducibility of results obtained with them, our approach provides a convenient method to recover precious experimentally modified cell lines from infection through the application of exogenous double-stranded RNA (dsRNA) targeting the DAV sequence. The in vitro transcribed dsRNAs were applied to infected S2 cells via direct “soaking,” utilizing the cells’ natural endocytic uptake. **Results**: We monitored treatment efficacy via RT-PCR-based detection of viral sequences, and our results demonstrate that continued application of DAV-specific dsRNAs led to a reduction in viral abundance and all eight tested cell lines appeared DAV-negative within several weeks of treatment. While a set of cell lines exhibited viral recurrence several weeks after the cessation of treatment, others remained virus-free for at least 16 weeks, suggesting that a permanent “cure” can indeed be achieved. Notably, while RNAi activation effectively cleared DAV in some cell lines, no beneficial effect was observed for a concomitantly applied *Drosophila* X-virus (DXV) treatment in the co-infected cultures. **Conclusions**: This suggests that DXV has more efficient evasion mechanisms but cannot protect DAV *in trans*. Our protocol thus provides a robust, scalable approach for clearing persistent DAV infections, but may not be effective against all viruses known to infect *Drosophila* cell cultures.

## 1. Introduction

RNA interference (RNAi) is a highly conserved immune response mechanism in eukaryotes that provides an efficient defense against viruses and transposable elements. Briefly, double-stranded RNA (dsRNA) is cleaved by Dicer (DCR) into small double-stranded fragments (siRNA) of 21 to 24 nt in length with a 2 nt single-stranded overhang on each 3′ end. After loading of the siRNA onto an effector protein from the Argonaute (Ago) family, one of the strands is released, and the bound siRNA guide remains single-stranded. The mature RNA-induced silencing complex (RISC) can thus bind and cleave complementary mRNAs to expose the resulting fragments to exonucleolytic degradation [1]. Originally identified as a tool to silence gene expression in *Caenorhabditis elegans* [2], RNAi has since become a versatile technique across diverse organisms and for the development of therapeutics [3,4,5].

*Drosophila melanogaster* has been an established model organism for over a century [6]. It is known that several viruses, including the RNA viruses DCV, DXV, DAV and Nora virus, can infect both natural and laboratory *Drosophila* populations and *Drosophila*-derived cell lines [7]. The RNAi pathway serves as an important antiviral mechanism [8], but independent resistance mechanisms have been described [9,10,11]. For *Drosophila* A virus (DAV), null mutations of RNAi pathway genes did not further increase the susceptibility of flies to infection when the virus was inoculated artificially by pricking [11]. It is, however, still unclear whether this is also true for natural infection routes with presumably lower inoculation doses or in cultured *Drosophila* cells.

Since virus infection of *Drosophila* cell cultures often has mild cytopathic consequences, an infection may remain undetected for a long time. Once discovered, however, a persistent virus infection of cell culture stocks needs to be addressed: Does this influence the outcome of ongoing and past experiments? Will it influence, e.g., the long-term viability of the cells, recovery of frozen stocks, or transfection and/or selection efficiencies? Clearly, the most desirable situation is to return to an uninfected cell culture system. Unfortunately, this is a laborious approach if genome-edited or stably selected cell lines need to be re-created from the uninfected original cell population.

Here, we present both evidence for a clear antiviral effect of the RNAi system on DAV in S2-cells and a cost-effective method to suppress DAV infection by activating the host RNAi machinery. Technically, this approach involves generating in vitro transcription templates by PCR-amplifying cDNA from a virally infected sample with virus-specific primers extended by the T7 promoter sequence. The PCR product is then transcribed in vitro (IVT) using T7 RNA-Polymerase, and the RNA is annealed to form dsRNA. If the cell line is competent for RNAi by “soaking”, this dsRNA can subsequently be applied periodically to the infected cell culture population. For example, many S2 cell line isolates efficiently take up the dsRNA without any transfection reagent via scavenger–receptor-mediated endocytosis [12]. However, the dsRNA cannot be applied in selection medium containing the antibiotic G418 [13]. The duration of the treatment will nonetheless need to be maintained over a period of weeks to months to permanently clear the infection. While this does take time, it does not involve a lot of handling steps once the dsRNA has been generated.

## 2. Results

**Figure 1:**
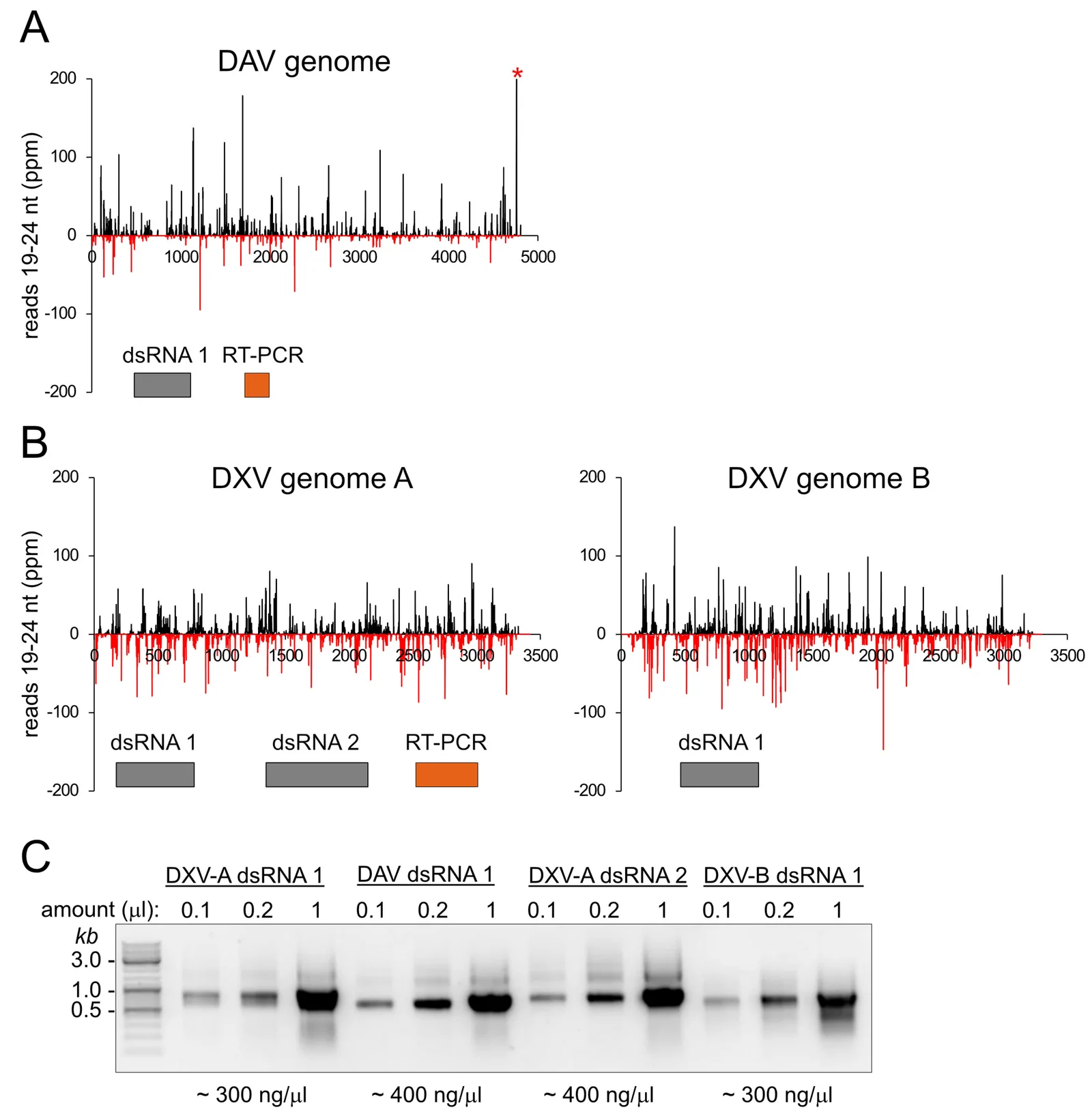
Persistent DAV and DXV infection of cultured cells. (**A**) Virus-derived siRNA reads from deep sequencing mapped to the *Drosophila* A virus (DAV) genome; sense reads are indicated in black and above the axis, antisense reads in red and below the axis. We used the position of the 3′-end for the sense reads and the 5′-end for the antisense reads. The normalization of sequencing depth was to all *Drosophila* genome matching reads and is expressed as parts per million (ppm). Asterisk: This signal was truncated for the diagram because an exceptionally high read number was mapped to this position (3346 ppm, this is reduced to 856 if only 21 + 22 mers are considered). The region targeted with the dsRNA fragment is shaded in gray on the bottom; the region amplified via RT-PCR for virus detection is shaded in orange. (**B**) Virus-derived siRNA reads were mapped to the *Drosophila* X virus (DXV) genome as described in (**A**); note that the scale is identical. DXV has two genomic RNAs, referred to as A (left) and B (right). The region targeted with the dsRNA fragment is shaded in gray on the bottom; the regions amplified via RT-PCR for virus detection are shaded in orange. (**C**) Analysis and yield estimation of the dsRNA fragments obtained after in vitro transcription (IVT). We estimated the yields by comparing the intensity of the 0.1 µL sample band to the more intense marker bands (labeled); these correspond to ~50 ng of DNA. While this approach is only a rough estimation, it is not affected by remaining NTPs from the transcription reaction.

We noticed a persistent infection of cell culture lines with DAV as well as *Drosophila* X-virus (DXV) by mapping small RNA sequencing libraries against a collection of *Drosophila* infecting virus genomes [14,15] (Figure 1A,B). Since the endogenously triggered RNAi pathway cannot apparently sufficiently affect the virus transcripts or genome efficiently, we wondered whether an artificial “boost”, created by providing exogenous dsRNA corresponding to the DAV genome, could provide a substantial benefit to the cells (Figure 1, targeted regions shaded in A and B). Note that we did not assess whether the cells are essentially all infected or whether our cultures are a mix of infected and non-infected cells.

To set up our detection and treatment protocol, we thus used cells that were persistently infected with DAV and DXV. We extracted RNA, transcribed it in vitro into cDNA, and used this as a template to create the dsRNA template amplicons. Additionally, we used the cDNA as a positive control sample for virus detection via PCR.

We generated dsRNA fragments targeting DAV (one fragment) and DXV (two fragments for the A genome part, one fragment for the B genome part), which led to four synthetic dsRNA fragments in total. Figure 1C shows the agarose gel of the final dsRNA preparation used to treat the viral infection. A dilution series was prepared to estimate the IVT yield in comparison to the marker bands. Based on the 1:10 diluted samples, we estimated the concentrations of the DXV-A fragment 1 to be 300 ng/µL, the DAV fragment 1 to be 400 ng/µL, the DXV-A fragment 2 to be 400 ng/µL, and the DXV-B fragment 1 to be 300 ng/µL. Note that a direct A_260_ measurement in the photometer can be quite misleading if unincorporated NTPs from the transcription reaction remain in the dsRNA preparation. We therefore prefer an estimation based on gel electrophoresis.

**Figure 2:**
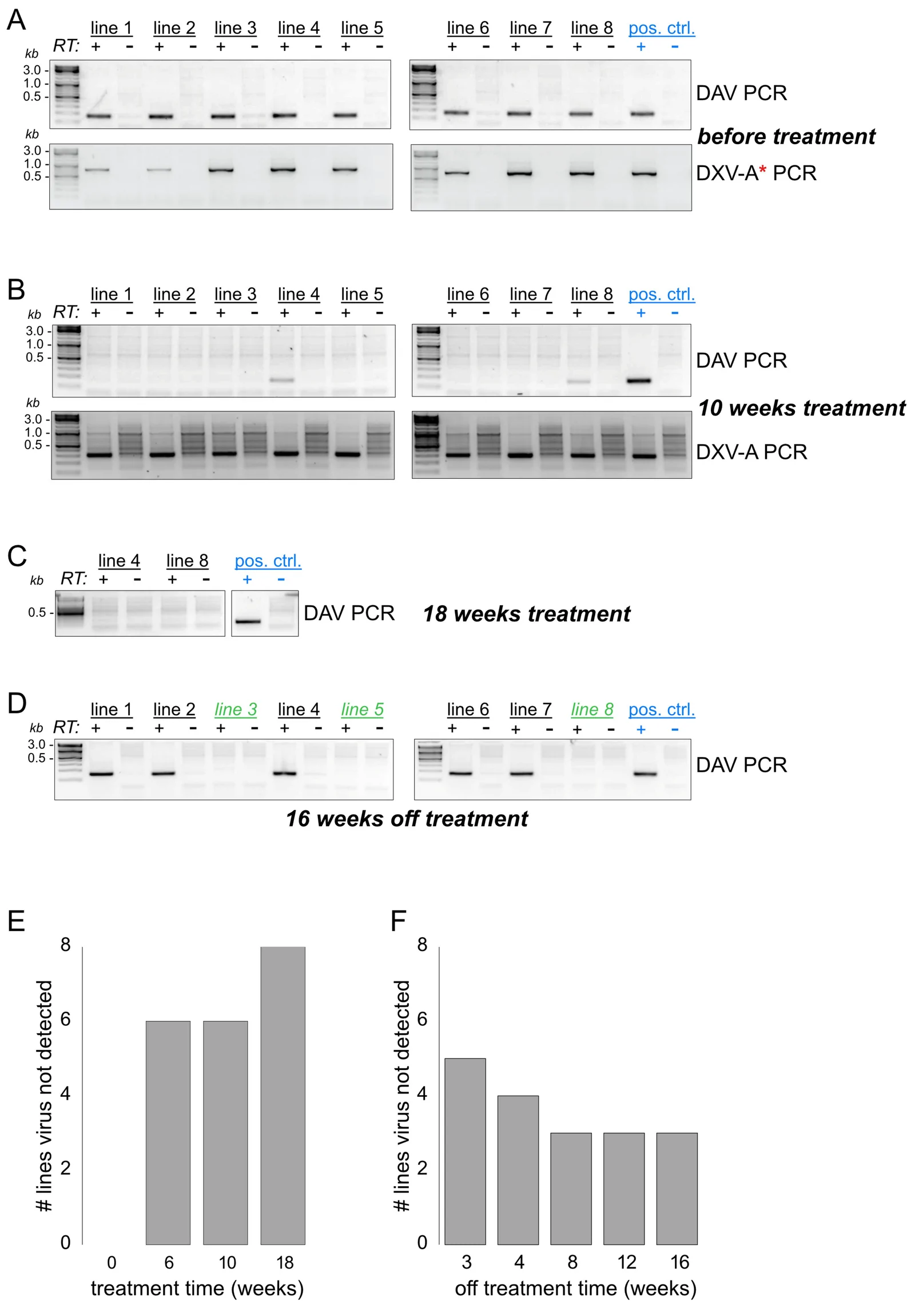
Determination of virus infection state via RT-PCR. (**A**) Before adding dsRNA, all eight cell lines harbored viral RNA that was detectable via RT-PCR. Here, we used the same primers for DXV detection that also served to generate the in vitro transcription template (hence labeled DXV-A* PCR). (**B**) After 10 weeks of dsRNA treatment, the majority of the cell lines were DAV-negative. However, the DXV infection status remained unchanged. After adding dsRNA, a different primer pair outside of the targeted region must be used (labeled DAV-A; see Section 4 for details). (**C**) After a total of 18 weeks of treatment, the remaining two cell lines had also reached undetectable levels of DAV RNA. (**D**) After 16 weeks of culturing without dsRNA treatment, the DAV infection had re-emerged in 5 out of 8 cell lines, but 3 lines (labeled in green) remained DAV-free. (**E**) Bargraph of the proportion of DAV-free cell lines during different time points of the treatment. (**F**) Bargraph showing the re-emergence of DAV infection at different time points after the end of the treatment.

A panel of eight infected S2 cell lines was treated over a time of six weeks. These cells were genetically modified to express different truncated versions of the *Drosophila* Zinc-finger protein Putzig (*pzg*). They were derived in the context of an independent research approach not related to viral infection and are thus not described further here. During the treatment period, we split the cells once per week at a ratio of 1:10 in a 48-well plate and treated them with 2 µg/mL (final concentration for each fragment) of all four dsRNA fragments. It is convenient to prepare a master mix of the dsRNAs for this. After six weeks of treatment, the cell lines were tested for the presence of DAV via RT-PCR, and six out of eight cell lines appeared negative. For the remaining two cell lines, the treatment had to be continued for another 12 weeks, thus lasting 18 weeks in total, until our RT-PCR approach no longer detected the DAV presence; an intermediate test after 10 weeks still demonstrated DAV presence (Figure 2B). From weeks 10 to 18, the DXV infection was treated only with DXV-A fragment 1 dsRNA (the other fragments were used up, and there was no indication for any treatment success with DXV anyways).

Following the apparently successful DAV treatment, all cell lines were maintained in medium without any dsRNA added, and we tested them every three to four weeks for re-emergence of DAV infection. As early as three weeks after the treatment had stopped, one third of the cell lines were again DAV-positive when assayed via RT-PCR. After 8 weeks, about two thirds of the cell lines showed re-emergence of the virus. The remaining three DAV-negative cell lines, however, then remained virus-free for 16 weeks after treatment; we stopped the observation period after this (Figure 2D).

## 3. Discussion

We present evidence that experimental activation of the RNAi pathway can efficiently reduce the viral load of a persistent DAV infection. This demonstrates that while RNAi can reduce the viral presence and may even fully clear the virus in cultured cells, the intrinsic RNAi response to DAV is insufficient. We did not determine true viral titers via limited dilution and cytopathology assays, but our RT-PCR analysis suggests abundant DAV transcripts and/or genomes in the infected cultures. Apparently, the dsRNA from the virus—a replication intermediate—is either of very low abundance in the cells (we cannot distinguish between replicating genome and viral mRNA with our PCR) or well shielded from Dicer-2 processing into siRNAs, or both. Furthermore, the successful activation of RNAi with exogenous dsRNA demonstrates that any potentially present anti-RNAi activities by the viruses (DAV and DXV in our cells) do not fully ablate the capacity to make and deploy virus-targeting siRNAs. During initial infection, there may thus be a window of opportunity where the cells can prevent the establishment of viral persistence and instead clear the infection. We have not directly tested whether the siRNA pathway lowers viral load once persistence of DAV has been established (i.e., without exogenously added dsRNA). This would require the inhibition of the RNAi pathway either by RNAi itself, where the dsRNA trigger would then compete for loading into Ago2 with antiviral silencing, or by genome editing, which likely favors adaptation mechanisms during the required clonal selection. This question is therefore best addressed by infecting mutant and control flies, which is beyond the scope of the current study.

We have only analyzed the infection on the level of the cell population, rather than testing single cells. It is thus conceivable that our cell populations represent a mixture of infected and uninfected cells that co-exist in the same dish. Considering that the cultures had already been maintained for weeks to months to allow for our desired expression of Putzig protein fragments (transfection, antibiotic selection, selection of stable integrations, and/or clonal knock-ins), it is hard to imagine that a substantial proportion of the cells in the culture dishes would have escaped the virus infection. Nonetheless, it is formally possible that the induced RNAi lowers the viral load in infected cells and thus reduces the probability of infecting the naïve cells in the same culture. In this case, the treatment would not correspond to a real “cure” but rather reduce the viral pressure on uninfected cells.

Reducing the DAV RNA levels below the detection limit of our assay (25 PCR cycles) required a treatment time of 6 to 18 weeks. This is non-negligible but the cultures do not require a lot of handling during that time: we split them once per week and added the dsRNA from a master stock solution. However, it is possible that the cultured cell population shifts some of its properties during this prolonged time in culture. We consider our approach most valuable for highly edited cell lines (e.g., double-tagged) that have already been cultured for many population doublings to introduce and select the modifications. Nonetheless, we advise that essential features of the cell cultures should be compared before and after the apparent viral clearance. The absence of virus may also change the cellular physiology (after all, this is the motivation to reduce the infection). A deliberate re-infection of the “cured” cultures may thus be necessary to reveal the virus-induced changes and separate them from the effects of prolonged culture. This obviously requires a strict hygiene protocol but we have successfully (and unsuspectingly) cultured cells with and without DXV/DAV infection using the same hood and the same incubator. We have no indication that the infection has spread from one culture into the next (e.g., due to aerosols in the pipettes). Conditioned cell culture medium, if needed (e.g., for single-cell cloning), must of course be derived from non-infected cultures. As a matter of fact, we suspect that our cells became infected when a supplement derived from live flies (“fly juice”) was added to the medium to improve growth with a sub-optimal serum batch.

When the treatment was stopped and cells were cultured without dsRNA in the medium, we saw the re-emergence of DAV after ~4 weeks in six out of eight cultures. This is presumably due to remaining viral particles at the end of the treatment and indicates a limited sensitivity of our RT-PCR detection. We have not tested whether the modification of our treatment procedure, such as more frequent splitting and/or washing cells with saline before each split, will improve the proportion of full viral clearance.

Concurrently with the successful DAV suppression, we did not notice *any* beneficial effect of activating RNAi against *Drosophila* X-virus (DXV). The most parsimonious explanation is that the two viruses may have distinct anti-RNAi effectors: While in DAV there could be, e.g., a shielding of the replicating (=dsRNA) genome, which can be circumvented by adding exogenous dsRNA, the resistance of DXV to such treatment (and the arguably more abundant siRNAs to begin with, compare Figure 1A,B) argues for alternative mechanisms. Interestingly, though, this must be a cis-effect limited to the DXV RNAs since DXV did not concomitantly protect the DAV transcripts present in the same cells. Conversely, the successful shielding (in case that is indeed the mechanism) of the DAV dsRNA from dicing does not spread to the DXV genome. Future research may reveal the distinct molecular mechanisms of protection from the host siRNA system in these (somewhat involuntary) model systems of insect virus/host interactions.

## 4. Materials and Methods

### 4.1. Small RNA Sequencing and Analysis

RNA was extracted from a stably cas9-expressing cell line [16] with Trizol. Small RNAs were gel-purified as previously described [17], and sequencing libraries were prepared with NEB (Ipswitch, MA, USA) Next kit E7300G and sequenced on an Illumina (San Diego, CA, USA) NextSeq 1000 System in-house. Adapter trimming, selection of 19–24 nt long reads, mapping, and visualization were performed as previously described [17]. To screen for viral siRNAs, we converted a published collection of *Drosophila* virus genome sequences [14] to a genome index using bowtie-build [18]. Thus, each viral genome represents a different “chromosome” for mapping, and this is a suitable input format for our downstream analyses to produce the sequence traces shown in Figure 1.

### 4.2. Oligonucleotide Sequences

The oligonucleotides used for the amplification of the in vitro transcription templates were DAV_dsRNA_sense_1 (5′-TAATACGACTCACTATAGGGAGAAACAGATGGGCCGGACAACG-3′), DAV_dsRNA_antisense_1 (5′-TAATACGACTCACTATAGGGAGACGTCGCCATAACAGACAACCCG-3′), DXV-A_dsRNA_sense_1 (5′-TAATACGACTCACTATAGGGAGACGTAAACAGCGCCCAGACCATC-3′), DXV-A_dsRNA_antisense_1 (5′-TAATACGACTCACTATAGGGAGAGATGTCAACTGGCCCTACGGAG-3′), DXV-A_dsRNA_sense_2 (5′-TAATACGACTCACTATAGGGAGAAGTGATGACCTTGGCCGACTAC-3′), DXV-A_dsRNA_antisene_2 (5′-TAATACGACTCACTATAGGGAGAGTCGTCTGCTGCATAAATGGCA-3′), DXV-B_dsRNA_sense_1 (5′-TAATACGACTCACTATAGGGAGAATAAGCCAGGCCATGAGGTCAC-3′), and DXV-B_dsRNA_antisense_1 (5′-TAATACGACTCACTATAGGGAGAGGAGAATTGGTCTGCTGCCCTC3′); the T7 promoter sequence is underlined. The oligonucleotides used to PCR-amplify DAV and DXV for detection purposes were DAV_detection_sense (5′-AGAACGGCACGCCAAAGAAC-3′), DAV_detection_anitsense (5′-CTGGCTTCGTTCCGGTTTGC-3′), DXV_detection_sense (5′-ACCGGCGAAAGAAGGCAAAG-3′), and DXV_detection_anitsense (5′-CGAGGTGGTATTGGCTGGGA-3′).

### 4.3. Synthesis of dsRNA for Treatment

Total RNA was extracted from 100 µL of resuspended cells with a urea-based extraction protocol [19]. Subsequently, cDNA was synthesized from ~3 µg of total RNA with the help of random hexamer primers (1 µM final concentration) and the RevertAid enzyme according to the supplier’s instructions (Thermo Fisher Scientific, Waltham, MA, USA). We also included an -RT sample to verify that the amplification product is indeed derived from reverse-transcribed RNA. PCR was then carried out with Taq polymerase under standard reaction conditions (2 mM MgCl_2_, annealing temperature of 50 °C for DAV product A and 62 °C for DAV product B and DXV products A and B, extension of 1 min. at 72 °C) and 1 µL of 1:5 diluted cDNA as template. The in vitro transcription reactions were performed as previously published [20], and we assume that corresponding, commercially available kits will function well in this context. After the transcription reaction, the magnesium-pyrophosphate precipitate was removed via centrifugation. We adjusted the supernatant to 50% isopropanol in order to precipitate and thus further concentrate the RNA, and then washed the pellet twice in 70% ethanol. Note that this does not reliably remove all non-incorporated NTP’s from the transcription reaction. After dissolving the RNA in a suitable volume of RNase-free water, we annealed the strands by placing the tube in a heating block at 94 °C and then letting it cool to room temperature by switching the block off. Since free NTPs may still be present, we quantified the dsRNA via agarose gel electrophoresis. In total, 1 µL each of pure dsRNA, a 1:5 dilution, and a 1:10 dilution were loaded on a non-denaturing 1.5% Agarose gel, and the band intensity was estimated by comparing with a DNA marker; the expected product size is between 600 and 800 nt.

### 4.4. Treatment of Cells

Cell culture was performed in standard Schneider’s medium supplemented with 20% FBS and Pen/Strep. Beyond that, no conditioned medium or fly extracts were added. The DAV and DXV infected S2 cells were seeded in a 48-well plate at a density of 1 × 10^6^ cells/mL. The virus-targeting dsRNAs were directly added to the culture medium at a final concentration of 2 µg/mL. After an incubation time of one week at 25 °C, the cells were split 1:10 into fresh cell culture medium, and the dsRNA was applied as before.

### 4.5. Monitor DAV and DXV Infection via PCR

We amplified cDNA (prepared as described above) with standard Taq polymerase using the DAV and DXV detection primers (see above), 3.5 mM MgCl_2_, and an annealing temperature of 61 °C for 25 cycles (DAV) or 30 cycles (DXV). To demonstrate that bona fide viral RNA was amplified, we included samples where the reverse transcriptase enzyme had been left out during the cDNA synthesis step (-RT controls). The reaction products were loaded on a 1.5% agarose gel, and the products of expected size (~150 nt for DAV, ~300 nt for DXV) were documented.

## 5. Conclusions

Persistent DAV infection of valuable *Drosophila melanogaster* cell culture lines can potentially be cured via the application of corresponding, in vitro synthesized dsRNA. The treatment needs to be applied over a period of at least 10 weeks, and potential recurrence of DAV must be monitored via RT-PCR. Our study demonstrates that the DAV RNA is susceptible to RNAi-mediated degradation and thus raises the mechanistic question why the endogenous RNAi response to DAV is sub-optimal.

## Data Availability

We submitted the sequencing data under accession number PRJEB113838, sample ERS30362210, to the European Nucleotide Archive (ENA).

## Abbreviations

The following abbreviations are used in this manuscript:

DAV	*Drosophila* A virus
DXV	*Drosophila* X virus
IVT	In vitro transcription
RT	Reverse transcriptase
PCR	Polymerase chain reaction
